# Clinical and cost-effectiveness analysis of an open label, single-centre, randomised trial of spinal cord stimulation (SCS) versus percutaneous myocardial laser revascularisation (PMR) in patients with refractory angina pectoris: The SPiRiT trial

**DOI:** 10.1186/1745-6215-9-40

**Published:** 2008-06-30

**Authors:** M T Dyer, KA Goldsmith, SN Khan, LD Sharples, C Freeman, I Hardy, MJ Buxton, PM Schofield

**Affiliations:** 1 Health Economics Research Group, Brunel University, Uxbridge, Middlesex, UK; 2 MRC Biostatistics Unit, Cambridge, UK; 3 Research and Development Unit, Papworth Hospital NHS Trust, Papworth Everard, Cambridge, UK; 4 Department of Cardiology, Papworth Hospital NHS Trust, Papworth Everard, Cambridge, UK; 5 Department of Anaesthetics, Papworth Hospital NHS Trust, Papworth Everard, Cambridge, UK; 6 Papworth Hospital NHS Trust, Papworth Everard, Cambridge, CB3 8RE, UK

## Abstract

**Background:**

Patients with refractory angina have significant morbidity. This study aimed to compare two of the treatment options, Spinal Cord Stimulation (SCS) and Percutaneous Myocardial Laser Revascularisation (PMR) in terms of clinical outcomes and cost-effectiveness.

**Methods:**

Eligible patients were randomised to PMR or SCS and followed up for exercise tolerance time (ETT), Canadian Cardiovascular Society (CCS) classification and the quality of life measures SF-36, Seattle Angina Questionnaire and the EuroQoL at 3, 12 and 24 months. Utilities were calculated using the EQ-5D and these and costs were compared between groups. The incremental cost-effectiveness ratio (ICER) per QALY for SCS compared to PMR was also calculated.

**Results:**

At 24 months post-randomisation, patients that had SCS and PMR had similar ETT (mean difference 0.05, 95% CI -2.08, 2.18, p = 0.96) and there was no difference in CCS classification or quality of life outcomes. The difference in overall mean costs when comparing SCS to PMR was GBP5,520 (95% CI GBP1,966 to GBP8,613; p < 0.01) and the ICER of using SCS was GBP46,000 per QALY.

**Conclusion:**

Outcomes after SCS did not differ appreciably from those after PMR, with the former procedure being less cost-effective as currently applied. Larger studies could clarify which patients would most benefit from SCS, potentially increasing cost-effectiveness.

**Trial registration:**

Current Controlled Trials ISRCTN09648950

## Background

There are estimated to be 30,000–50,000 new patients with refractory angina pectoris per year in Europe, who are unsuitable for conventional revascularisation [1]. Procedures aiming to improve quality of life in affected patients include transmyocardial laser revascularisation (TMR) and percutaneous myocardial laser revascularisation (PMR). TMR uses laser ablation to create transmural channels in ischaemic myocardium via a thoracotomy whilst the less invasive PMR, delivered via catheter, creates channels from the endocardium partially through the myocardium. Previous studies comparing TMR and optimal medical management have shown improved relief of angina offset by perioperative mortality and morbidity [2,3]. One UK trial-based analysis concluded that the technology, with an incremental cost per quality-adjusted life year gained (QALY) over 12 months of over £200,000, was not cost-effective in comparison to optimal medical management [4]. Published clinical evidence suggests that PMR is an attractive alternative to TMR due to significantly lower procedural mortality and morbidity [5,6]. One UK trial-based analysis of PMR versus medical management produced an estimate over 12 months of over £50,000 per QALY [7], again above currently accepted UK thresholds.

Spinal cord stimulation (SCS) has been used for many years in the treatment of chronic pain and, since 1995, for the treatment of refractory angina pectoris. SCS is a surgically implanted device that produces a low voltage electrical impulse near the dorsal surface of the spinal cord, which blocks pain stimuli, leaving the patient with paraesthesia instead. Observational and randomized studies of SCS have found a reduction in angina frequency and an improvement in quality of life whilst not preventing nor concealing the symptoms of myocardial infarction [8-13]. Retrospective data from small, uncontrolled studies and one prospective study in patients with severe angina have shown that the higher costs of initial SCS treatment may be offset by fewer subsequent hospital admissions [13-18]. This study aims to assess the cost-effectiveness of SCS relative to PMR up to 24 months post-procedure using prospectively collected data from a randomised controlled trial in a UK setting.

## Methods

Full details of the randomised trial, including detailed description of the two procedures, baseline characteristics, and outcome measures are reported elsewhere [18]. Between December 2000 and December 2003, 68 patients in a tertiary referral centre for cardiovascular disease were randomised to either SCS with optimal medical therapy (n = 34) or PMR with optimal medical therapy (n = 34). Patients were followed up to endpoints at 3, 12 and 24 months. This report focuses on the 24 month results. Approval was obtained from the local research Ethics Committee prior to study commencement and informed consent was obtained.

The primary objective of the study was to compare the effect of SCS versus PMR on exercise treadmill time using a modified Bruce Protocol at 24 months post-treatment. Secondary measures of effectiveness included angina (as measured by the Canadian Cardiovascular Society classification), morbidity/mortality and quality of life, measured by the disease-specific Seattle Angina Questionnaire (SAQ) and the generic Short Form-36 (SF-36) and EuroQoL questionnaires. The SAQ measures functional status of patients with angina. SF-36 and EuroQoL are more general measures of health status. The SF-36 scores patients based on 8 broad health outcomes and allows for calculation of summary physical and mental health scores. EuroQoL scores mobility, self-care, usual activities, pain/discomfort and anxiety/depression and combines these with self-rated health status, to give a health status score. The EuroQoL is also used to generate a utility measure for use in cost-effectiveness analyses. The economic analysis was designed alongside the clinical study to estimate cost-effectiveness of SCS relative to PMR, up to 24 months.

### Statistical Analysis

Analysis was by intention to treat for subjects for whom follow-up data were available. Survival was summarized and compared using Kaplan-Meier methods and the log-rank test. Adverse events were compared using Poisson regression. Total exercise time was summarised using the mean and standard error. Analysis of variance was used to assess the difference in exercise time between the two groups adjusting for baseline time. Similar models were used to assess the difference in health-related quality of life scales. Since not all patients experienced angina on the treadmill, the mean time to angina was estimated from the area under a Kaplan-Meier curve, with angina-free patients censored at the total exercise time. The change in angina-free exercise time was calculated from the estimated means and standard errors, accounting for the correlation between baseline and follow up estimated from those patients who were not censored. The comparison between groups in angina-free exercise time used Student t-tests based on these Kaplan-Meier summaries. CCS class was compared between groups using the Mantel-Haenszel test for ordered categorical variables. Fisher's exact test was used to assess differences in the proportion of patients having a decrease of 2 or more CCS classes (considered clinically significant), medication usage and proportion of patients free from angina during exercise. Differences were considered significant if p < 0.05.

### Economic Analysis

An NHS perspective was adopted. Resources included were those associated with the procedures, cardiac-related medication, and cardiac or non-cardiac-related inpatient or outpatient admissions, up to 24 months after procedure. Resources consumed solely as a result of the research element of the trial protocol were excluded. All resources were valued at 2005/06 costs.

### SCS and PMR procedures

Both procedures were costed according to the most relevant elective inpatient Health Resource Group (HRG) finished consultant episodes [19]. The SCS procedure was costed according to either HRG A08: Percutaneous Image Controlled Pain Procedures or HRG R11: Spinal Cord Surgery. If a patient was admitted to hospital for two days or more they were assigned to the HRG R11 (n = 30) and for less than two days they were assigned to HRG A08 (n = 2). These HRG costs were based on standard resource use items including theatre and staff time, pathology, radiology and ECG tests. For both procedures, the cost of relevant procedure-related equipment was added to generate a total mean cost per patient. Patient specific data were collected on length of stay for the immediate period of hospitalisation and appropriate cardiac ward bed day costs were assigned. For further details of the main categories of resource use and costs, see table 1.

**Table 1:** Main resource use and cost categories

**Resource Use**	Cost applied*	**Unit Cost**	**Source**
**Procedures:**			

Cardiac Ward Bed day	P	£285	Papworth NHS Trust
SCS Equipment – Electrode/IPG	F	£8,240	Papworth NHS Trust
PMR Equipment – (Laser/Catheter)	F	£5,755	Papworth NHS Trust
SCS Procedure (HRG: A08)	F	£3,040	Papworth NHS Trust
SCS Procedure (HRG: R11)	F	£3,685	Papworth NHS Trust
PMR Procedure – Catheter Lab	F	£197	Papworth NHS Trust
Pathology/Radiology Tests (Both procedures)	P	£45	Papworth NHS Trust

**Cardiac-related medication (cost per month):**			

Calcium Antagonists -Atenolol (50 mg)	P	£0.90	BNF 2006^17^
Long-Acting nitrates – Imdur (60 mg)	P	£12	BNF 2006
Short-Acting nitrates – GTN spray (1200 mcg)	P	£1.40	BNF 2006
ACE Inhibitors – Lisinopril (10 mg)	P	£1.80	BNF 2006
Diuretics – Frusemide (40 mg)	P	£1.10	BNF 2006
Statins – Simvastatin (20 mg)	P	£1.60	BNF 2006

*P = patient, F = fixed

### Cardiac-related medication

Use of major cardiac-related medication were recorded for all patients. Since information on dosage was not available, an average dosage based on clinical opinion was applied and a monthly cost was calculated (see Table 1). If a patient stopped taking a drug between follow up visits, it was assumed that cessation occurred at the mid-point between the visits. Costing to 24-months was carried out using the British National Formulary [20].

### Inpatient and outpatient episodes

Length of stay and diagnosis were recorded for all inpatient episodes. For cardiac-related inpatient episodes, the mean national cost of the relevant HRG finished consultant episode was applied, adjusted for actual length of stay [19]. Papworth hospital costs of overnight admissions for cardiac-related investigations were adjusted to a national level by the National Reference Cost Index. Cardiac-related inpatient episodes for crossover patients (two SCS and four PMR) during the 12 – 24 month follow-up period were included. The mean national cost of the relevant HRG finished consultant episode was applied for non-cardiac-related inpatient episodes.

### Patient Utility and QALYs

At baseline, and 3, 12 and 24 months post-procedure, patients completed the EuroQol questionnaire. Each patient's self-reported classification was valued according to the tariff of UK population values by Dolan et al. [21]. Assuming a linear change in values between time points, patient specific utilities up to 24 months from randomisation were calculated. For missing EQ-5D data, values were interpolated between adjacent visits. Where 24-month data were missing, the value from the last visit was carried forward. A utility value of zero was applied to patients who died.

The QALYs to 24 months from randomisation were calculated as the area under the utility curve to 24 months or time of death. General linear modelling was used to adjust follow-up utility values for baseline. To estimate confidence intervals, without assuming any parametric form for cost and QALY distributions, bootstrapping was used (1000 samples) [22]. Costs and utilities incurred between 12 and 24 months were discounted at an annual rate of 3.5% based on the latest NICE guidance [23]. As a small number of patients in both treatment groups were censored prior to 24-month follow-up, published methods to deal with censored data were applied to the cost and QALY estimates [24]. The Incremental Cost-Effectiveness Ratio (ICER) was calculated as the difference in mean cost divided by the difference in mean QALY. Uncertainty due to parameter estimation was demonstrated by calculation of the cost-effectiveness acceptability curve. This curve plots the probability that SCS is cost-effective, compared with PMR, against the maximum societal willingness to pay for 1 QALY gain.

## Results

### Recruitment and follow-up

**Figure 1:**
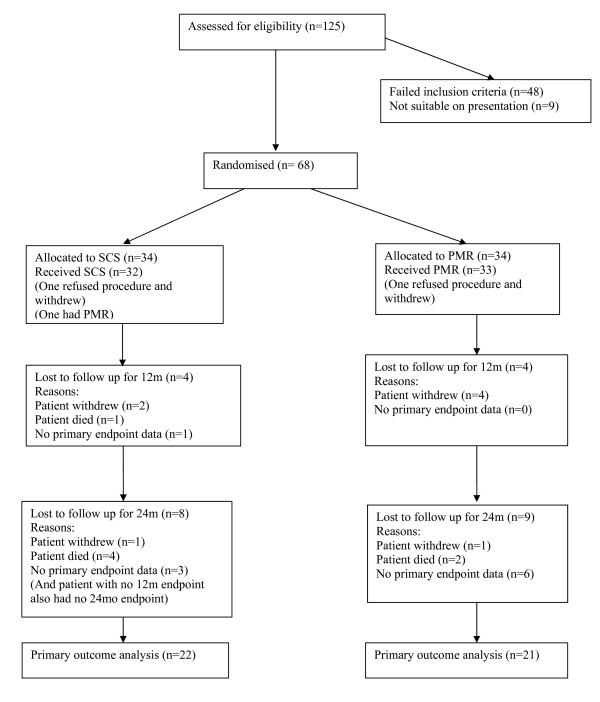
CONSORT diagram showing the flow of participants through the trial comparing SCS with PMR.

Trial methods and clinical results for the first 12 months have been published [18]. From 125 patients assessed for eligibility, sixty-eight patients were recruited and randomised (Figure 1). At baseline, patients in the treatment groups had similar mean age (64.2 vs 62.9 years), sex distribution (85% vs 91% male), CCS class (Class III: 65% vs 74%; Class IV: 35% vs 26%) and anti-anginal medication. During 24 months of follow up, of the patients allocated to SCS, five died, three withdrew from the trial, three were unable to do the 24 month exercise test, and one test was terminated due to equipment malfunction (3 patients provided quality of life data), leaving 22 with exercise test data. Of the patients allocated to PMR, two died during follow up, 5 withdrew from the trial (1 later died more than 24 months post-randomization) and 6 could not perform an exercise test at 24 months (4 of whom provided quality of life data), leaving 21 patients with exercise test data at 24 months (see Figure 1 for further details). Resource use data were available for all participants.

### Adverse events

There were eight deaths to the end of September 2005, five in the SCS group and three in the PMR group (one patient had previously withdrawn). Causes of death (days post procedure) were ischaemic heart disease (18), metastatic squamous cell carcinoma (442), presumed malignancy (630) and two acute MI (589 and 660) in the SCS group and stomach carcinoma (430), ischaemic heart disease/MI (490) and unknown (683) in the PMR group. Survival was 85% at 24 months in the SCS group and 94% at 24 months in the PMR group (p = 0.46).

Between randomization and procedure, one SCS patient had a bout of unstable angina requiring hospitalization. Another patient experienced a bent lead during SCS implantation, which was classed as an event of moderate severity. In the first year post-procedure, there were 75 non-fatal, adverse events in 32 patients and in the second year there were 53 in 30 patients (Table 2). The SCS group reported more adverse events than the PMR group in the first year but less in the second year (relative rates [RR] 1.5, 95% CI 0.9, 2.3, p = 0.10 and 0.8, 95% CI 0.5, 1.4, p = 0.44). Over the 24-month period, 36 events in the SCS group and 41 events in the PMR group were disease-related (RR 0.9, 95% CI 0.6, 1.3, p = 0.51). There were 25 SCS related events up to 24-months, four of which occurred in patients who had crossed over from PMR. There were four events related to the PMR procedure up to 24 months, one of which occurred in a patient that had crossed over. Of all the adverse events, 62 in the SCS group and 54 in the PMR group were classed as severe (RR 1.1, 95% CI 0.8, 1.6, p = 0.53) (Table 2).

**Table 2:** Adverse events *

	Months 0–12 post- procedure		Months 13–24 post- procedure	
		
	SCS	PMR	SCS	PMR
Disease-related				
Unstable angina	16 (10)	16 (7)	7 (5)	12 (4)
Myocardial infarction	4 (3)	1 (1)	1 (1)	1 (1)
Loss of pain relief/angina worse	5 (5)	3 (2)	3 (3)	8 (6)
Total disease related	25 (18)	20 (10)	11 (9)	21 (11)
SCS related: Infection of SCS system	0	NA	0	2 (2)
Undesirable change in stimulation	7 (5)	NA	5 (5)	2 (1)
Pain at neurostimulator site	5 (3)	NA	0	0
Neurostimulator migration	2 (2)	NA	1 (1)	0
Lead migration	1 (1)	NA	0	0
PMR related: Femoral pseudoaneurysm	0	1 (1)	0	0
Groin haematoma	1 (1)	2 (2)	0	0
Other	4 (3)	7 (7)	7 (5)	4 (4)
Total	45 (17)	30 (15)	24 (18)	29 (12)
Total excluding SCS/PMR related	29 (14)	27 (14)	18 (12)	25 (11)
**Severity**				
Mild	2 (2)	2 (2)	0	0
Moderate	5 (5)	0	0	3 (2)
Severe**	38 (15)	28 (15)	24 (18)	26 (11)
Total	45 (17)	30 (15)	24 (18)	29 (12)

*Data are number of events (number of patients), NA means Not Applicable.

**Severe included events which: required hospital admission and/or surgery, prolonged hospital stay, or were life-threatening or fatal.

### Exercise Tolerance Tests and CCS class

The results from the exercise tolerance tests are summarised in Table 3. Adjusting for baseline, there was little difference in exercise time between the two groups at 24 months (Table 3). Mean time to angina was also similar in the two groups.

**Table 3:** Comparisons between SCS and PMR in mean exercise tolerance and CCS at 24 months

	*SCS*	*PMR*	Difference adjusted for baseline 95%CI	p
Exercise test at 24 months	(n = 22)	(n = 21)		
Mean (SEM) exercise time	7.04 (0.76)	7.70 (0.88)	0.05 (-2.08, 2.18)	0.96
Mean* (SEM) time to angina	7.69 (1.08)	7.57 (1.08)	0.91 (-2.67, 4.49)	0.62
No angina during exercise	11 (50%)	7 (33%)		
CCS class at 24 months	(n = 22)	(n = 21)		
1	3 (14%)	2 (9%)		0.55**
2	9 (41%)	9 (43%)		
3	8 (36%)	6 (29%)		
4	2 (9%)	4 (9%)		
Change in CCS >= 2 classes				
No	15 (68%)	17 (81%)		0.49**
Yes	7 (32%)	4 (19%)		

* Calculated from area under Kaplan-Meier time to angina curves (some patients stopped exercising before onset of angina). Comparisons based on change from baseline and within patient correlation estimated from uncensored patients from both groups combined (ρ = 0.28).

**p-values unadjusted for baseline

At 24 months there was no significant difference between the treatment groups in CCS class. A greater number of SCS patients had a decrease of two or more CCS classes, although this result was not statistically significant.

### Health-related quality of life

**Figure 2:**
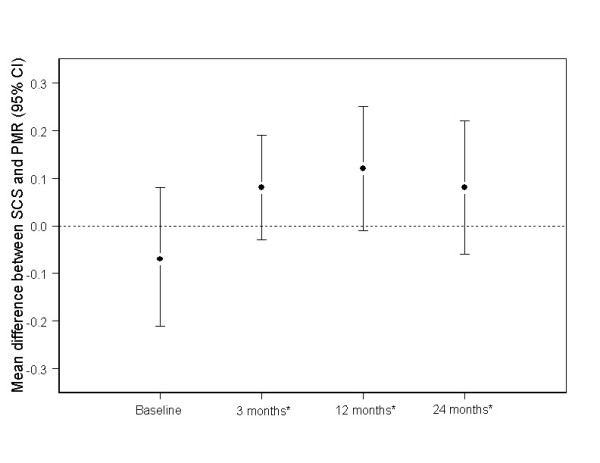
**Mean difference between SCS and PMR in EQ-5D scores at 3, 12 and 24 months post-procedure (values above 0 favour SCS)**. * Adjusted for baseline.

**Figure 3:**
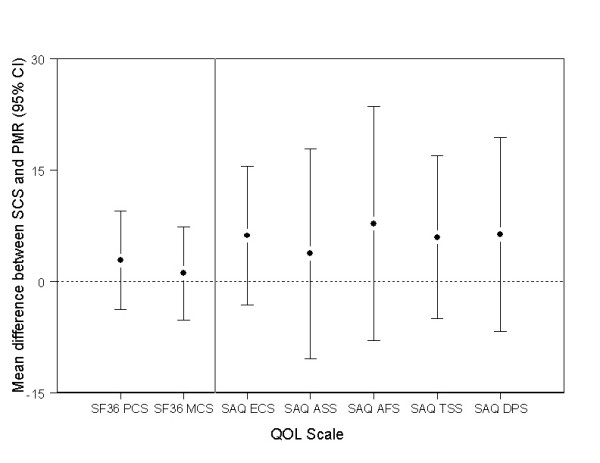
**Mean difference between SCS and PMR in SF-36 and SAQ scales at 3, 12 and 24 months post-procedure, adjusted for baseline scores (values above 0 favour SCS)**. SF36 – 36-item short form survey; PCS – physical component score; MCS – mental component score; SAQ – Seattle Angina Questionnaire, ECS – Exertional capacity scale; ASS – Anginal stability scale; AFS – Anginal frequency scale; TSS – Treatment satisfaction scale; DPS – Disease perception scale.

Figures 2 and 3 show the mean difference between groups in health-related quality of life measures at 3, 12 and 24 months adjusting for baseline. For most scales, both groups reported improvements from baseline (data not shown), but there were no significant differences between the groups at follow up (all p ≥ 0.08, Figures 2 &3).

### Medication

As at baseline, all patients were being treated with maximally tolerated anti-anginal medication at 24 months. Among the patients who completed the exercise test, there were no significant differences between SCS and PMR groups in use of Beta Blockers (91% vs. 81%), Calcium Antagonists (68% vs. 71%), ACE Inhibitors (46% vs. 57%), long acting Nitrates (77% vs. 91%) short acting Nitrates (100% vs. 86%) Aspirin (77% vs. 86%) or Nicorandil (77% vs. 91%) (all p ≥ 0.11).

### Resource use and costs

Information on resource use and costs to 24 months is presented in Table 4.

**Table 4:** Mean costs and QALYs to 24 months post-randomisation

**Resource**	SCS (n = 34) Mean cost per patient (95% CI)	PMR (n = 34) Mean cost per patient (95% CI)	Mean cost difference (95% CI)
PMR/SCS Equipment*	£8,239	£5,755	
PMR/SCS Procedure	£4,353 (£4,046 to £4,397)	£326 (£307 to £353)	
PMR/SCS LOS	£756 (£685 to £872)	£812 (£695 to £988)	
Total SCS/PMR procedure cost	£13,350 (£13,112 to £13,491)	£6,892 (£6,757 to £7,087)	£6,459 (£6,196 to £6,642) †
**Non-procedural cost**	£957 (£839 to £1,088)	£1,025 (£891 to £1,147)	
Cardiac-related medication	£3,294 (£2,076 to £5,844)	£3,645 (£1,638 to £7,537)	
Cardiac inpatient episodes	£97 (£22 to £281)	£146 (£18 to £492)	
Non-cardiac inpatient episodes	£4,350 (£3,064 to £6,873)	£4,811 (£2,750 to £8,633)	
**Total non-procedural costs**			-£461 (-£4,399 to £1,964) ‡
Overall treatment costs at 24 months**	£17,736 (£16,398 to £19,202)	£12,215 (£9,603 to £15,448)	£5,520 (£1,966 to £8,613)†

**Utility**	SCS (n = 34)	PMR (n = 34)	QALY difference
	Mean QALY per patient (95% CI)	Mean QALY per patient (95% CI)	(95% CI)

QALYs up to 24 months**	1.19 (1.040 to 1.319)	1.07 (0.960 to 1.178)	0.12 (-0.04 to 0.30)‡

*Fixed cost applied to all patients; **Overall costs and QALYs taking account of censoring; † p < 0.01; ‡ p > 0.1

### Initial Procedure Costs

The mean cost per patient of the PMR (laser equipment and catheter) and SCS (implantable pulse generator, electrode) equipment was £5,755 and £8,239 respectively. Other resources used during the procedure (staff time, overheads, catheter lab, theatre time etc) were much greater for SCS (£4,353 versus £326), mainly because it was a longer procedure (two to three hours on average) which takes place in theatre (based on British Pain Society recommendations for best clinical practice [25]) whilst the shorter PMR procedure (90 minutes) takes place in the catheter lab. Length of hospital inpatient stay (and therefore cost of stay) was similar: mean 2.9 days for PMR and 2.7 days for SCS. The mean total procedure cost was £6,892 (95% CI £6,757 to £7,087) for PMR and £13,350 (95% CI £13,112 to £13,491) for SCS.

### Follow-up Costs

The costs of cardiac-related medication and cardiac and non-cardiac inpatient episodes, including subsequent SCS/PMR procedures, were similar (Table 4). The mean overall difference between the two groups for non-procedural costs favoured SCS but was not significant at -£461 (95%CI -£4,399 to £1,964).

### Overall costs SCS vs. PMR

The difference in overall mean costs to 24 months of £5,520 (95% CI £1,966 to £8,613; p < 0.01) indicates that the additional cost of the SCS procedure was not offset by the small reduction in non-procedure costs over two years.

### Utility

There were no significant differences in overall utility at 24 months between groups after adjustment for baseline (Table 4). There was a mean QALY difference favouring SCS of 0.12 (95% CI: -0.04 to 0.30; p > 0.1). This difference equates to approximately 6 weeks in perfect health gained from the use of SCS over PMR (95% CI: -2 weeks to 16 weeks).

### Cost-Utility

**Figure 4:**
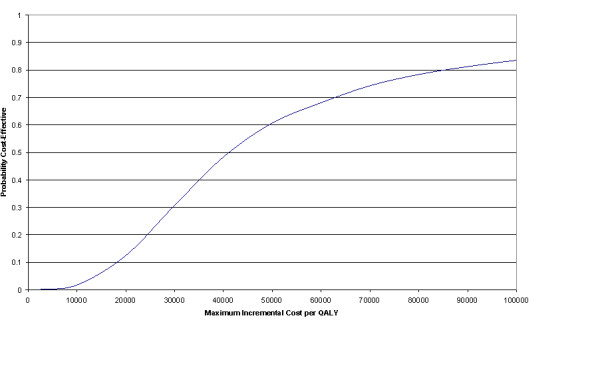
Cost-effectiveness acceptability curve.

The mean incremental cost per QALY of using SCS was £46,000 over 24 months. Figure 4 shows the cost-effectiveness acceptability curve. Taking £30,000 per QALY as the maximum acceptable in the UK, which is broadly in line with NICE guidance [26,27], the probability of SCS being cost-effective compared with PMR over a two year time period is approximately 30%.

### Sensitivity Analysis

This was a relatively small trial taking place within a single centre, resulting in imprecise estimates of costs and outcomes. To assess the impact of some of the trial parameters and the results' relevance to other settings, sensitivity analyses were conducted including:

(i) the effect of lower capital cost of the SCS equipment or more intensive use (i.e. higher patient throughput) by halving SCS equipment costs to £4,120 per case. This resulted in a smaller difference in mean costs (£2,340) and a lower ICER of £19,500 per QALY.

(ii) the effect of carrying out SCS in the catheter lab instead of in theatre, reducing total cost of the procedure by approximately £2,500. Similarly, this results in a lower difference in mean cost (£2,262) and a lower ICER of £18,850 per QALY.

(iii) combining (i) and (ii) resulted in SCS being cheaper than the PMR strategy (by approximately £1,700) and more effective.

(iv) sensitivity of the results to deaths was explored. Omission of these deaths (5 SCS; 2 PMR) results in a slightly higher mean QALY difference in favour of SCS of 0.19 reducing the ICER to approximately £30,000 per QALY.

(v) the SF-36 allows calculation of an alternative utility measure (SF-6D) [28,29], resulting in mean QALY difference favouring SCS of 0.011 (95% CI: -0.12 to 0.13; p > 0.1), and a much higher incremental cost per QALY of approximately £500,000.

## Discussion

### Clinical Results

This study compared two treatments for refractory angina and reported the results of follow-up at 24 months. SCS patients had greater time to angina and lower CCS class at 3 months, but these differences were not maintained at 12 months [18], nor at 24 months, as shown here. It was thought that differences between SCS and PMR groups might increase over time as follow up continued, but the differences between the groups were smaller at 24 months (0.05 minutes in exercise time and 0.91 minutes to angina versus 0.59 and 1.23 at 12 months).

Past studies have shown PMR to be a safer and more cost-effective option than TMR. It was thought that the increased costs of SCS might be offset by decreased morbidity and hospital admission costs compared to PMR. Patients did have non-significant improvements in exercise and quality of life outcomes with SCS over PMR, but these were small and not statistically significant, and costs were higher for SCS patients. In addition, while SCS is less invasive than TMR, it is more invasive than PMR.

### Economics

The additional NHS cost of using SCS versus PMR was estimated to be £5,520. When combined with the small health benefits observed, the mean incremental cost per QALY was £46,000. Since this is based on early experience within a specialist tertiary centre, results may well improve with greater experience over time. Exploring the cost-effectiveness of SCS versus PMR in the first and second half of the study suggested there was an improvement over time, which could be indicative of a learning curve effect. For patients recruited during 2000/01, the ICER was estimated at £230,000 per QALY (95% CI: -£2,670,000 to £590,000) whereas for 2002/03, the ICER was estimated at £18,000 per QALY (95% CI: -£21,000 to £51,000). This improvement can largely be explained by better outcomes, in terms of survival and QoL, experienced by SCS patients in the second half of the study. Outcomes may not, however, be as good in a non-specialist centre.

A limitation is that the small sample size in this study resulted in low precision, in particular in the case of the QALY estimates, which showed no significant difference between the two groups. Given that we did not observe a difference, there is no a priori reason to assume that the relative cost-effectiveness of the two procedures would change if observed over a longer period.

Even if the (most optimistic) ICER estimate of £30,000 per QALY is the most appropriate, the issue of how SCS fits in to the overall picture in terms of cost-effectiveness of interventions for treatment of refractory angina is not clear. In this study, SCS was compared to PMR as the next best treatment to estimate cost-effectiveness. In a previous cost-effectiveness analysis, PMR was compared against optimal medical management producing an ICER over 12 months of over £50,000 per QALY [7], a figure well above the current maximum thresholds in the UK. Here, SCS was compared to PMR rather than optimal medical management thus raising the question of whether SCS was compared with the next best alternative treatment, at least from the cost-effectiveness perspective. Given that neither TMR nor PMR were cost-effective over 12 months in comparison with optimal medical management, one might presume that SCS is unlikely to prove cost-effective against medical management. This should be confirmed by comparing SCS with medical management either directly within a randomised controlled trial, or indirectly via economic modelling. In addition, we saw lower clinical effectiveness here than has been seen in other studies, however, this is likely due to over-estimation of the effectiveness of treatments in observational studies due to selection bias.

## Conclusion

The results suggest that there is little difference between SCS and PMR with respect to clinical and patient outcomes. Larger studies of SCS are needed to determine which patients benefit most from SCS; targeting the treatment to these patients could make SCS more cost-effective.

## Competing interests

This study was sponsored by Medtronic SA, who were responsible for funding trial related investigations (perfusion scans, treadmill tests), research staff for data collection and travel expenses for patients.

## Authors' contributions

MTD performed data analysis and wrote the manuscript. KG performed data analysis, and contributed to writing the manuscript. SK managed patients and contributed to writing the manuscript. LS designed the study, performed data analysis and contributed to writing the manuscript. CF managed the trial, managed data, and contributed to writing the manuscript. IH designed the study, gave patients SCS treatment, managed patients, and contributed to writing the manuscript. MB designed the study, performed data analysis and contributed to writing the manuscript. Peter Schofield designed the study, gave patients PMR treatment, contributed to writing the manuscript, acted as principal investigator, and acted as guarantor for this study.
